# Safety and Efficacy of Pulsed Field Ablation for Atrial Fibrillation in Elderly Patients

**DOI:** 10.1002/joa3.70267

**Published:** 2026-01-02

**Authors:** Federico Follesa, Alix Prévot, Samy Gribissa, Xavier Waintraub, Marine Thuillot, Nicolas Badenco, Guillaume Duthoit, Estelle Gandjbakhch, Mikael Laredo

**Affiliations:** ^1^ Sorbonne Université, Unité de Rythmologie, Institut de Cardiologie, AP‐HP, Hôpital Universitaire Pitié‐Salpêtrière Paris France

**Keywords:** Ablation safety, Aging, Atrial Arrhythmia, Catheter ablation, Pentaspline catheter

## Abstract

**Background:**

Pulsed‐field ablation (PFA) is increasingly used for catheter ablation of atrial fibrillation (AF), but older patients remain underrepresented in clinical trials. This study aimed to compare procedural outcomes and mid‐term effectiveness of PFA in patients aged ≤ 75 and > 75 years.

**Methods:**

In this retrospective single‐center cohort, 479 consecutive patients underwent PFA for AF between January 2022 and April 2024. Patients were grouped by age (≤ 75 vs. > 75 years at ablation). Procedural parameters and acute complications were compared. Arrhythmia‐free survival was assessed with Kaplan–Meier analysis after an 8‐weeks blanking period, and predictors of recurrence were evaluated using Cox regressions.

**Results:**

Of 479 patients (mean age 65.0 ± 12.1 years; 73.6% males), 104 (21.7%) were > 75 years at ablation. Patients > 75 years had more comorbidities, including hypertension and impaired renal function. Pulmonary vein isolation was achieved in 99.8% of cases. Acute complication rates were similar between groups (7.7% in > 75 vs. 8.5% in ≤ 75, *p* = 1.00), with low rates of tamponade (1.5%) and stroke (1.3%). Kaplan–Meier analysis showed no difference in arrhythmia‐free survival. At 6 months, freedom from atrial arrhythmia was 81.4% in the > 75 group and 83.8% in the ≤ 75 group (*p* = 0.57); corresponding rates at 12 months were 60.1% and 68.6%. Age was not an independent predictor of recurrence. At last follow‐up, 75.7% of patients were off antiarrhythmic drugs.

**Conclusions:**

PFA in patients > 75 years is associated with low complication rates and favorable rhythm outcomes, comparable to those in younger patients. These findings support the use of PFA in elderly patients with AF.

## Graphical abstract

Pulsed‐field ablation (PFA) was evaluated in 479 patients with atrial fibrillation (AF), 52% paroxysmal. Among 104 patients > 75 years, complication rates and 6‐ and 12‐month arrhythmia‐free survival were not significantly different from younger patients. Age did not predict recurrence. PFA appears safe and effective in elderly AF patients.

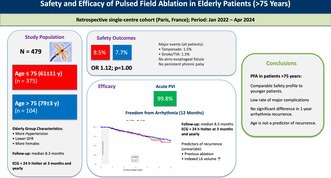

## 1 Introduction

Atrial fibrillation (AF) is a public‐health epidemic, affecting around 60 million individuals worldwide, with an age‐specific surge that peaks after the seventh decade of life [1]. In Europe, this demographic shift caused AF to be described as a “geriatric syndrome” and advocating early rhythm‐control to limit heart‐failure admissions, stroke, and cognitive decline [2]. Unfortunately, conventional pharmacological rhythm‐control remains poorly tolerated in older adults, who are often burdened by polypharmacy, multimorbidity, and heightened pro‐arrhythmic or bleeding risk [3].

Catheter ablation has therefore emerged as a cornerstone therapy [4], yet outcome data in patients beyond 75 years reveal a ≥ 24% higher arrhythmia‐recurrence rate and a 64% excess of major complications using radiofrequency or cryoballoon ablation [5]. Age‐related atrial fibrosis, esophageal fragility and reduced cardiorespiratory reserve underscore the importance of limiting thermal lesions and shortening procedure times in this vulnerable population [6, 7, 8]. Pulsed‐field ablation (PFA) delivers ultra‐short, high‐voltage electrical pulses that create myocardial‐selective electroporation while sparing the esophagus, phrenic nerve and pulmonary veins [9]. In the pivotal ADVENT trial, PFA has been proved non‐inferior to radio‐frequency or cryoballoon ablation for freedom from atrial tachyarrhythmia and procedure‐related serious adverse events at 1 year [10]. Real‐world data from the EU‐PORIA registry have subsequently confirmed a favorable safety profile and short procedure times across more than 1.200 cases [11]. Despite these encouraging signals, patients older than 75 years remain markedly under‐represented in PFA trials. Emerging data from registries such as EU‐PORIA [12], ATHENA [13], and FARADISE [14] indicate that PFA is generally safe in elderly patients, with acute outcomes comparable to younger individuals, although age may still influence long‐term arrhythmia recurrence. However, these studies included relatively small numbers of elderly patients or heterogeneous clinical profiles, and long‐term follow‐up remains limited. The present study was therefore designed to compare procedural metrics, acute safety and mid‐term rhythm outcomes between patients aged ≤ 75 and > 75 years undergoing PFA in a high‐volume center. By addressing further this knowledge gap, we aim to optimize rhythm‐control strategies for an expanding segment of the AF population.

## 2 Methods

### 2.1 Study Design

This is a retrospective observational cohort study conducted at the cardiology department of the Pitié‐Salpêtrière Hospital in Paris, France. Clinical data were retrieved from the local electronic health record system. The study complies with the reference methodology MR004 of the French National Commission for Informatics and Liberties (CNIL) and was conducted in accordance with the Declaration of Helsinki. All patients provided written informed consent for the anonymized use of their routine clinical data for research purposes. The authors had full access to the data and assume full responsibility for their integrity.

### 2.2 Study Population

The study population consisted of all consecutive adult patients who underwent catheter ablation for AF using a PFA system since the introduction of the technique from January 2022 to April 2024. All patients met European Society of Cardiology guidelines indications for catheter ablation [2]. Two groups were formed according to age at ablation: ≤ 75 years and > 75 years. Left atrial (LA) dimensions were obtained primarily from pre‐procedural computed tomography scans, or from transthoracic echocardiography when not available.

### 2.3 Ablation Procedure

All patients underwent PFA using the FARAPULSE platform (Boston Scientific, Marlborough, MA) under general anesthesia. Echo‐guided femoral venous access was obtained, and transseptal puncture was performed under transesophageal echocardiographic guidance. A 31‐ or 35‐mm pentaspline FARAWAVE catheter was used at the operator's discretion under fluoroscopic guidance [9]. All PFA procedures aimed at achieving PVI, using a standard protocol previously described. In brief, four applications per PV in basket and flower conformations were delivered with a ∼36° rotation after each two applications, with a minimum of eight applications per PV. Additional applications on the PV antra were performed as deemed necessary by the operator. Extra‐PV ablation was performed on the LA posterior wall and/or mitral isthmus at the operator's discretion. PVI was confirmed by entrance and exit block using the Farawave catheter at each pulmonary vein ostium. All procedures were performed under periprocedural uninterrupted anticoagulation using direct oral anticoagulants or vitamin K antagonists.

### 2.4 Outcomes and Follow‐Up

The primary safety outcome was the occurrence of acute procedural complications, including: pericardial tamponade, neurovascular complications such as stroke or transient ischemic attack (TIA), post‐procedural chest pain, acute pulmonary edema, and vascular complications related to femoral venous access (e.g., hematoma, pseudoaneurysm, arteriovenous fistula). The primary outcome was the recurrence of any sustained (lasting ≥ 30 s) atrial arrhythmia, 6 and 12 months after ablation. An 8‐week blanking period was used. All patients were followed up locally. Follow‐up included at least one outpatient visit 3 months after the procedure including an ECG and a 24 h Holter‐ECG, followed by a yearly outpatient visit including a 24 h Holter ECG.

### 2.5 Statistical Analysis

Continuous variables are presented as mean ± standard deviation or median with interquartile range (IQR), depending on distribution assessed with the Shapiro–Wilk test. Categorical comparisons used the Pearson chi‐square or Fisher exact test, while continuous variables were analyzed with the Student *t* test or Wilcoxon test. Group comparisons relied on ANOVA or Kruskal–Wallis tests, selected according to variable distribution. For multiple pairwise comparisons, Tukey's or Steel–Dwass procedures were applied to control for alpha inflation. Time‐dependent analysis of the effectiveness outcome was assessed using the Kaplan–Meier method, and the Log‐Rank test assessed differences between groups. Cox proportional hazards regression identified factors linked to atrial arrhythmia recurrence, providing hazard ratios (HRs) with 95% confidence intervals (CIs). Variables with *p* ≤ 0.10 in univariate analysis were entered into a multivariable model using forward selection. All analyses were two‐sided, with *p* < 0.05 considered statistically significant. All analyses were performed using RStudio (v2024.04.2, Boston, MA).

## 3 Results

### 3.1 Baseline Characteristics

A total of 479 patients underwent catheter ablation of AF using pulsed field ablation between January 2022 and April 2024: 375 in the ≤ 75‐years group and 104 in the > 75‐years group. Baseline characteristics are detailed in Table 1. Female sex was significantly more represented in the > 75‐years group (49.0% vs. 26.4%, *p* < 0.001). In addition, elderly patients had a lower height (1.67 ± 0.09 m vs. 1.74 ± 0.10, *p* < 0.001), lower weight (70.7 ± 14.9 kg vs. 81.6 ± 17.2, *p* < 0.001), and lower BMI (25.2 ± 4.7 vs. 26.7 ± 4.9, *p* = 0.006). The ethnic distribution was comparable between the two groups. Hypertension was significantly more prevalent in the > 75‐years group (55.8% vs. 42.1%, *p* = 0.02), while diabetes mellitus, coronary artery disease, prior myocardial infarction, heart failure, and valvular disease showed no statistically significant differences. Dyspnea was the most frequent symptom in both groups, with a higher prevalence in older patients (52.9% vs. 44.5%), followed by palpitations. Patients > 75 years more frequently fell into classes NYHA II and III (48.1% and 23.1%, respectively), with a tendency toward worse functional status. EHRA classification showed a significantly greater proportion of class 3 in the elderly group (25.0% vs. 17.3%, *p* = 0.02). The prevalence of paroxysmal and persistent AF was balanced between groups. Renal function was more frequently impaired in older patients, with 42.3% having an eGFR < 60 mL/min (vs. 19.5%, *p* < 0.001). The left ventricular ejection fraction was slightly higher in older patients (58.4% ± 9.8 vs. 55.3% ± 12.4, *p* = 0.04). LA volume did not differ significantly across groups. Beta‐blocker use was less frequent in older patients (50.0% vs. 66.4%, *p* = 0.003), while flecainide was more frequently prescribed in younger patients (24.8% vs. 10.6%, *p* = 0.003). Anticoagulation therapy was widely adopted in both groups, with over 89% of patients receiving direct oral anticoagulants.

**TABLE 1:** Baseline characteristics of the study population according to age group.

	All (*n* = 479)	> 75‐years group (*n* = 104)	≤ 75‐years group (*n* = 375)	*p*
Age, years	65.0 ± 12.1	78.8 ± 3.2	61.2 ± 10.8	< 0.001
Male sex (%)	329 (68.7)	53 (51.0)	276 (73.6)	< 0.001
Height, m	1.7 ± 0.1	1.67 ± 0.09	1.74 ± 0.10	< 0.001
Weight, kg	79.3 ± 17.3	70.7 ± 14.9	81.6 ± 17.2	< 0.001
Ethnicity (%)				
Caucasian	410 (85.6)	96 (92.3)	314 (83.7)	0.18
North African	33 (6.9)	4 (3.8)	29 (7.7)	
Afro‐Caribbean	20 (4.2)	2 (1.9)	18 (4.8)	
Asian	16 (3.3)	2 (1.9)	14 (3.7)	
BMI, kg/m^2^	26.4 ± 4.9	25.19 ± 4.7	26.72 ± 4.9	0.006
Hypertension (%)	216 (45.1)	58 (55.8)	158 (42.1)	0.02
Diabetes mellitus (%)	57 (11.9)	10 (9.6)	47 (12.5)	0.521
Coronary artery disease (%)	95 (19.8)	25 (24.0)	70 (18.7)	0.282
Myocardial infarction (%)	42 (8.8)	13 (12.5)	29 (7.7)	0.18
History of heart failure (%)	113 (23.6)	27 (26.0)	86 (22.9)	0.61
Valvular heart disease (%)	30 (6.3)	8 (7.7)	22 (5.9)	0.65
Symptoms (%)				0.21
None	24 (5.0)	3 (2.9)	21 (5.6)	
Fatigue	25 (5.2)	6 (5.8)	19 (5.1)	
Palpitations	198 (41.3)	36 (34.6)	162 (43.2)	
Chest pain	10 (2.1)	4 (3.8)	6 (1.6)	
Dyspnea	222 (46.3)	55 (52.9)	167 (44.5)	
NYHA functional class (%)				0.05
I	157 (32.8)	24 (23.1)	133 (35.5)	
II	206 (43)	50 (48.1)	156 (41.6)	
III	82 (17.1)	24 (23.1)	58 (15.5)	
IV	34 (7.1)	6 (5.8)	28 (7.5)	
EHRA classification (%)				0.02
1	135 (28.2)	21 (20.2)	114 (30.4)	
2	241 (50.3)	57 (54.8)	184 (49.1)	
3	91 (19)	26 (25)	65 (17.3)	
4	12 (2.5)	0 (0)	12 (3.2)	
Type of atrial fibrillation (%)				
Persistent	230 (48)	54 (51.9)	176 (46.9)	0.43
Long‐standing persistent^a^	76 (31.8)	21 (37.5)	55 (30.1)	0.37
Persistent AF duration, months^b^	20.3 ± 45.6	27.2 ± 71.6	18.4 ± 35	0.46
Previous cardioversion (%)	143 (29.9)	32 (31.1)	111 (29.6)	0.87
Previous ablation procedures (%)	70 (14.6)	13 (12.5)	57 (15.2)	0.59
History of stroke or TIA (%)	44 (9.2)	10 (9.6)	34 (9.1)	1.00
History of COPD (%)	23 (4.8)	5 (4.8)	18 (4.8)	1.00
GFR, mL/min				< 0.001
> 60	358 (74.9)	60 (57.7)	298 (79.7)	
30–60	103 (21.5)	40 (38.5)	63 (16.8)	
< 30	10 (2.1)	3 (2.9)	7 (1.9)	
On dialysis	7 (1.5)	1 (1)	6 (1.6)	
CHADS‐VASc score (%)				< 0.001
0	73 (15.2)	0 (0)	73 (19.5)	
1	83 (17.3)	0 (0)	83 (22.1)	
2	122 (25.5)	13 (12.5)	109 (29.1)	
3	97 (20.3)	34 (32.7)	63 (16.8)	
4	68 (14.2)	35 (33.7)	33 (8.8)	
5	28 (5.8)	17 (16.3)	11 (2.9)	
6	7 (1.5)	4 (3.8)	3 (0.8)	
7	1 (0.2)	1 (1)	0 (0)	
Cardiac implantable device				0.24
Pacemaker	27 (5.6)	9 (8.7)	18 (4.8)	
ICD	21 (4.4)	3 (2.9)	18 (4.8)	
Medications				
Beta‐blockers (%)	301 (62.8)	52 (50)	249 (66.4)	0.003
Sotalol (%)	32 (6.7)	9 (8.7)	23 (6.1)	0.49
Flecainide (%)	104 (21.7)	11 (10.6)	93 (24.8)	0.003
Amiodarone (%)	166 (34.7)	43 (41.3)	123 (32.8)	0.13
Anticoagulant therapy (%)				0.15
DOAC	427 (89.1)	95 (91.3)	332 (88.5)	
VKA	27 (5.6)	7 (6.7)	20 (5.3)	
Heparin	2 (0.4)	1 (1)	1 (0.3)	
Echocardiographic parameters^c^				
LVEF, %^c^	55.9 ± 11.9	58.36 ± 9.8	55.3 ± 12.4	0.04
LVEF > 40%/≤ 40% (%) ^c^				0.09
> 40%	338 (86)	74 (92.5)	264 (84.3)	
≤ 40%	55 (14)	6 (7.5)	49 (15.7)	
Left atrial volume, mL/m^2^ ^d^	48.4 ± 19.5	52.7 ± 18.1	47 ± 19.8	0.1

*Note:* Values are expressed as mean ± standard deviation (SD) or as absolute numbers (percentages).

Abbreviations: AF, atrial fibrillation; BMI, body mass index; COPD, chronic obstructive pulmonary disease; CT, computed tomography; DOAC, direct oral anticoagulant; EHRA, European Heart Rhythm Association; GFR, glomerular filtration rate; ICD, implantable cardioverter defibrillator; LVEF, left ventricular ejection fraction; NYHA, New York Heart Association; PM, pacemaker; TIA, transient ischemic attack; VKA, vitamin K antagonist.

^a^ Data available for 239 patients.

^b^ Data available for 190 patients.

^c^ Data available for 393 patients.

^d^ Data available for 180 patients.

### 3.2 Procedural Outcomes and Safety

Procedural characteristics are shown in Table 2. PVI was obtained in 478 (99.8%) patients, with no significant difference between groups. There was no significant difference in the number of PFA applications between groups, but PFA applications at the LA posterior wall were used more frequently in the > 75‐years group (37.5% vs. 25.1%, *p* = 0.017). There was no difference in procedural time, fluoroscopy time, and duration.

**TABLE 2:** Procedural and technical characteristics of the study population according to age group.

	All (*n* = 479)	> 75‐years group (*n* = 104)	≤ 75‐years group (*n* = 375)	*p*
Same‐day discharge (%)	65 (13.6)	9 (8.7)	56 (15)	0.14
Final cardioversion (%)	121 (27.4)	40 (41.2)	81 (23.5)	< 0.001
Cavotricuspid isthmus ablation (%)	87 (18.3)	24 (23.1)	63 (17)	0.20
Farawave catheter size (%)				0.70
31 mm	429 (89.7)	94 (91.3)	335 (89.3)	
35 mm	49 (10.3)	9 (8.7)	40 (10.7)	
Rhythm at procedure start (%)				0.05
Atrial fibrillation	147 (31.1)	42 (41.2)	105 (28.4)	
Atrial flutter	15 (3.2)	3 (2.9)	12 (3.2)	
Sinus rhythm	310 (65.7)	57 (55.9)	253 (68.4)	
Total number of PFA applications	53.3 (±17.9)	53.8 (±16.8)	53.2 (±18.3)	0.72
Extra‐PV applications^a^	21 (±11.7)	20.8 (±11.4)	21 (±11.8)	0.84
LA roof (%)	158 (33)	36 (34.6)	122 (32.5)	0.78
LA posterior wall (%)	133 (27.8)	39 (37.5)	94 (25.1)	0.017
Mitral isthmus (%)	37 (7.7)	7 (6.7)	30 (8)	0.82
PVI achieved (%)	478 (99.8)	104 (100)	374 (99.7)	1.00
Fluoroscopy time, min	22.3 (±10.8)	23 (±12.8)	22.1 (±10.2)	0.53
Radiation dose (cGy/cm^2^)	600.4 (±684)	649.8 (±835.2)	586.5 (±635.9)	0.49
Procedure duration, min	72.3 (±29.7)	74.1 (±36.3)	71.8 (±28)	0.90

*Note:* Values are expressed as absolute numbers (percentages) or as mean ± standard deviation, as appropriate.

Abbreviations: LA, left atrium; PFA, pulsed field ablation; PV, pulmonary veins.

^a^ Data available for 221 patients.

Overall, 40 patients (8.4%) experienced at least one complication (Table 3): 8 in the > 75‐years group (7.7%) and 32 in the ≤ 75 years group (8.5%), with no statistically significant difference. The odds‐ratio for any complication in the > 75 years group was 1.12 (95% CI 0.50–2.5, *p* = 1.00). Cardiac tamponade was observed in seven patients (2 in the > 75‐years group; 5 in the ≤ 75 years group, *p* = 0.65). All cases in the younger group were successfully managed by percutaneous drainage. In the > 75‐years group, one patient required immediate sternotomy. Complications related to vascular access included 12 patients with hematoma, 3 with pseudoaneurysm and 2 with arteriovenous fistulas. Fourteen of the seventeen vascular complications were managed conservatively, while three required percutaneous intervention. No significant differences were detected between groups either in incidence or management approach. Six patients experienced neurologic events (stroke or TIA), distributed evenly across the two age groups, with no significant difference. Other rare adverse events included three cases of acute pulmonary edema (1 in the > 75‐years group; 2 in the ≤ 75 years group, *p* = 0.52), all of which resolved with medical treatment.

**TABLE 3:** Procedural complications according to age group.

Complication	All (*n* = 479)	> 75‐years group (*n* = 104)	≤ 75‐years group (*n* = 375)	*p*
Any complication	40 (8.4%)	8 (7.7%)	32 (8.5%)	1.00
Acute pulmonary edema	3 (0.6%)	1 (1.0%)	2 (0.5%)	0.52
Cardiac tamponade	7 (1.5%)	2 (1.9%)	5 (1.3%)	0.65
Chest pain	7 (1.5%)	0 (0.0%)	7 (1.9%)	0.36
Neurovascular complications	6 (1.3%)	2 (1.9%)	4 (1.1%)	0.62
Vascular complications (total)	17 (3.5%)	3 (2.9%)	14 (3.7%)	0.84
Hematoma	12 (2.5%)	2 (1.9%)	10 (2.7%)	
Pseudoaneurysm	3 (0.6%)	1 (1.0%)	2 (0.5%)	
Arteriovenous fistula	2 (0.4%)	0 (0.0%)	2 (0.5%)	

*Note:* Values are expressed as absolute numbers (percentages).

### 3.3 Follow‐Up and Recurrence

**FIGURE 1:**
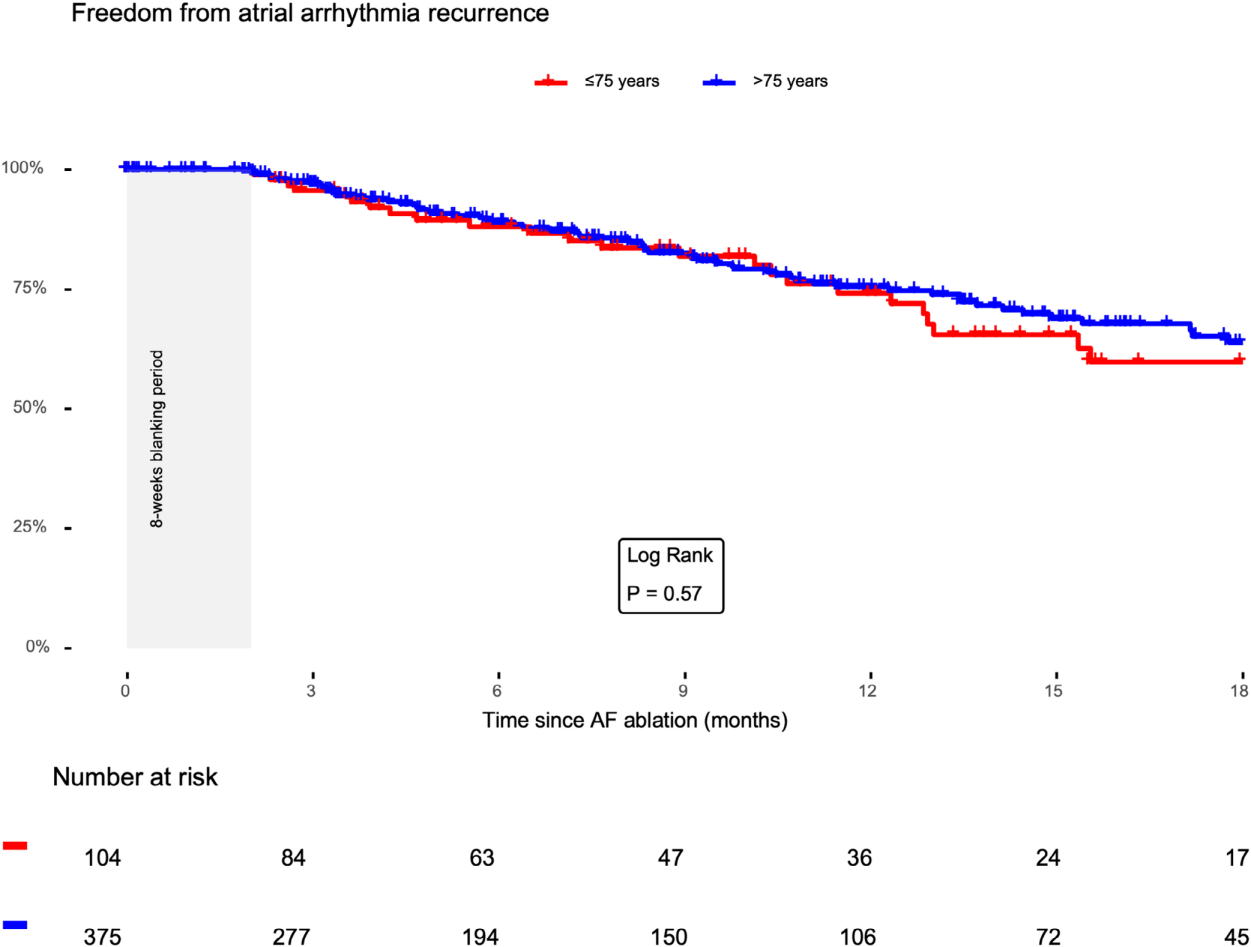
Freedom from atrial‐arrhythmia recurrence after PFA, according to age. Kaplan–Meier curves display the probability of remaining free from documented atrial‐arrhythmia recurrence in patients aged ≤ 75 years (red) and > 75 years (blue) following PFA of AF. Episodes occurring within an 8‐week blanking window after the procedure were not counted as recurrences.

Overall, the median follow‐up time was 8.3 (IQR 3.4–15.2) months. After a blanking period of 8 weeks, 95 (20.0%) patients experienced a sustained arrhythmia recurrence across both groups, 69 (18.4%) in the ≤ 75‐years‐group and 27 (26.0%) in the > 75‐years‐group. The cumulative probability of freedom from sustained arrhythmia recurrence across both groups was 83.2% (95% CI 79–87.7) at 6‐months follow‐up and 66.6% (95% CI 60.5–73.2) at 12‐months follow‐up. In the > 75‐years‐group, freedom from sustained arrhythmia recurrence was 81.4% (95% CI 72.7–91.2) at 6‐months follow‐up and 60.1% (95% CI 47.7–75.7) at 12‐months; in the ≤ 75‐years‐group, freedom from sustained arrhythmia recurrence was 83.8% (95% CI 79.1–88.8) at 6‐months follow‐up and 68.6% (95% CI 61.8–76.1) at 12‐months (Log‐Rank *p* = 0.57) (Figure 1).

Overall, at discharge after ablation, 153 patients (31.8%) were on beta‐blockers alone, 87 (18.1%) on amiodarone, 60 (12.5%) on flecainide, and 25 (5.2%) on sotalol. At the end of follow‐up, amiodarone was continued in 50 (10.4%) patients, flecainide in 42 (23.5%), and sotalol in 24 (5%). In total, excluding beta‐blockers, 364 patients (75.7%) were left without any anti‐arrhythmic at the end of follow‐up, 82 (78.8%) in the > 75‐years‐group and 282 (75.2%) in the ≤ 75‐years‐group (*p* = 0.52).

Previous ablations and indexed LA volume were significantly associated with recurrence. In a multivariable model, there was no variable independently associated with, although indexed LA volume showed a trend toward increased risk (HR 1.01 per 1 mL/m^2^ increase, 95% CI 1.00–1.02, *p* = 0.08) (Table 4).

**TABLE 4:** Variables associated with sustained atrial arrhythmia during follow‐up (Cox regressions).

	Univariable			Multivariable		
	HR	95% CI	*p*	HR	95% CI	*p*
Male	1.19	0.78–1.82	0.42			
Age (1 year increase)	1.02	0.99–1.04	0.06	1.00	0.98–1.03	0.86
Hypertension	1.35	0.91–2.02	0.14			
Persistent AF	1.19	0.79–1.78	0.39			
Previous ablations	1.90	1.11–3.26	0.02	1.48	0.60–3.67	0.40
Indexed LA (1 mL/m^2^ increase)	1.02	1.01–1.03	0.007	1.01	1.00–1.02	0.08
LVEF (1% increase)	0.99	0.98–1.02	0.85			
Procedure duration (1 min increase)	0.99	0.98–1.00	0.32			
AF/flutter at procedure start	1.08	0.71–1.64	0.70			
Extra‐PV applications	1.09	0.73–1.62	0.68			

Abbreviations: AF, atrial fibrillation; HR, hazard ratio; LA, left atrium; LVEF, left ventricular ejection fraction; PV, pulmonary veins.

Although procedure duration differed significantly between operators (*p* < 0.001), this variability did not translate into significant differences in procedural safety or acute effectiveness. There were no differences in complication rates across operators (*p* = 0.12), and freedom from sustained arrhythmia recurrence did not differ significantly (*p* = 0.42).

## 4 Discussion

The main results are that:

- PFA of AF yielded a low complication rate in patients older than 75 years, with no significant difference as compared to younger patients, despite more frequent comorbidities.
- There was no significant difference in the freedom from AF recurrence 6‐ and 12‐months after PFA in patients older than 75 years as compared to younger patients.
- Age was not significantly associated with AF recurrence.

The burden of AF in late life extends beyond stroke and heart failure: large cohort studies have linked incident AF with a substantially increased risk of cognitive decline and dementia [15]. Understanding whether safer, durable rhythm control may help slow this progression makes focused investigation in the ≥ 75 years population particularly relevant. Although the ADVENT and PULSED AF phase III trials confirmed noninferior efficacy and low serious adverse event rates, they excluded patients older than 75 years [10, 16]. As a result, there is limited evidence to guide rhythm control strategies in this important group.

Our single center cohort of 479 consecutive PFA procedures, 104 of which involved patients > 75 years old, adds to the emerging real‐world evidence.

### 4.1 Safety

In our cohort of 479 patients, the overall complication rate was 8.4%, which is higher than those reported in large multicenter registries of pulsed field ablation. This difference is probably due to the broader inclusion of minor adverse events in our analysis, such as vascular complications, chest pain, and acute pulmonary edema, which are not uniformly captured or reported across studies.

When focusing specifically on major complications, our results are consistent with the existing literature. In our study, cardiac tamponade occurred in 1.5% of patients, and stroke or TIA in 1.3%. By comparison, the MANIFEST‐PF registry reported a 1.9% rate of major complications, including 1.1% tamponade and 0.4% stroke [17]; EU‐PORIA documented 1.7% major events, with 1.1% tamponade and 0.6% cerebrovascular events [11]; while the ATHENA registry showed only 0.3% major events, comprising 0.2% tamponade and 0.1% TIA [13]. No cases of atrio‐esophageal fistula or persistent phrenic nerve injury were observed in our study, consistent with the safety profile observed across all three registries.

Our findings contrast with parts of the existing literature on PFA in elderly patients. In a sub‐analysis of the EU‐PORIA registry, PFA demonstrated similar 1‐year efficacy and overall complication rates in patients > 80 years compared with younger individuals, but strokes were more frequent in the elderly [12]. Given that this analysis included a moderate number of elderly patients (*n* = 88), a lack of power in our cohort is unlikely to explain the difference. Moreover, our single‐center registry spans the very early adoption phase of PFA, capturing the initial learning curve of all operators. The multicenter prospective ATHENA registry similarly reported an age‐independent safety profile, including in patients ≥ 75 years [13]. However, age remained an independent predictor of arrhythmia recurrence: individuals < 65 years experienced fewer and later recurrences than those ≥ 65 years at 1‐year follow‐up. Notably, older patients in ATHENA more often had persistent AF and were more frequently female—features not present in our population. In an interim analysis of the FARADISE registry, patients ≥ 75 years (*n* = 158) showed procedural times and acute outcomes comparable to younger patients, despite having more comorbidities and more frequent extra‐PV ablation [14]. Serious adverse events were uncommon and not significantly higher in the elderly, supporting comparable short‐term safety with PFA; long‐term follow‐up is pending. Adding to this growing body of evidence, our study shows that arrhythmia outcomes remain favorable in a larger cohort of patients > 75 years, with a longer 18‐month Holter‐based follow‐up.

When compared with thermal energy sources, PFA appears at least as safe in this age group. Cryoballoon ablation studies in elderly patients report complication rates around 6%–7%, with events including pericarditis, access‐site bleeding, and transient phrenic nerve palsy [18, 19] while RF cohorts [20, 21] show total event rates between 3% and 7%, with tamponade rates of 1.6% and direct comparisons between cryoballoon and RF in elderly patients confirming similar safety profiles [6, 22]. A multicenter analysis of 983 ablations in patients > 75 years (PFA, cryoballoon, RF) reported an overall 1% rate of major complications across all technologies, with no significant differences between techniques [23]. The absence of thermal injury mechanisms in PFA [24] likely explains the elimination of esophageal and phrenic complications observed both in our practice and in registries.

### 4.2 Effectiveness

Freedom from any atrial arrhythmia at the end of follow‐up was 80% in our population. Break down by age showed recurrence of 18% in patients ≤ 75 years versus 26% in those > 75 years (*p* = 0.12, not statistically significant). These values mirror the effectiveness result reported in multicenter PFA studies: EU‐PORIA documented a 74% arrhythmia‐free rate at 1 year [11], with a separate sub‐analysis reporting 70% arrhythmia‐free survival at 12 months in the same age group [12], while Dello Russo et al. [13] found a rate of 73.1% in a separate elderly cohort. These findings are consistent with those reported in the pivotal trials. The ADVENT trial showed a 1‐year success rate of 73.3% for PFA, comparable to 71.3% with conventional thermal ablation [10]. In the PULSED AF trial, success rates were 66.2% in paroxysmal and 55.1% in persistent AF [16].

Compared with thermal techniques, our success rate is comparable to cryoballoon ablation results in patients over 80 [18, 19], and consistent with radiofrequency studies specifically focused on the elderly. Okamatsu et al. observed an 82.9% arrhythmia‐free survival at 1 year in patients aged ≥ 80 years undergoing ablation index‐guided RF ablation [20]; similarly, Aldaas et al. found no significant difference in recurrence rates between patients ≥ 80 years and younger ones, with 52.4% of very elderly patients remaining arrhythmia‐free off antiarrhythmic drugs during long‐term follow‐up [21].

Propensity matched and registry data also demonstrate equivalent efficacy between cryoballoon and RF in this age group [6, 22]. Likewise, a large multicenter comparison of 983 ablations in patients > 75 years by Nakasone et al., comprising 221 PFA, 216 cryoballoon, and 546 RF procedures, showed 77% arrhythmia free survival with PFA at 1 year, statistically indistinguishable from cryoballoon (81%) and RF (75%) [23]. Importantly, the parallel performance across all three technologies reinforces the premise that in elderly patients substrate complexity rather than energy modality drives outcomes. Meta‐analyses suggest that arrhythmia recurrence after catheter ablation may be slightly higher in elderly patients; while some studies report non‐significant differences [5], a more recent analysis indicates a modest but statistically significant increase [25].

Extra‐PV lesions were performed at operator discretion and tailored to atrial size, comorbidities, frailty, and anticipated ability to tolerate redo procedures, with additional variability related to operator experience. Although adjunctive lesions—particularly posterior‐wall ablation—have not shown clear benefit for arrhythmia‐free survival [26], they remain common in practice and are easily performed with the pentaspline PFA catheter without added procedural risk [27]. Consistent with other multicenter PFA cohorts, older patients in our series more often underwent posterior or anterior wall ablation [14, 28]. Extra‐PV lesions were not associated with arrhythmia recurrence.

Taken together, these concordant results support the use of PFA in elderly patients without sacrificing safety or rhythm‐control efficacy.

Future studies should aim to confirm these findings through prospective, multicenter randomized trials enrolling patients ≥ 75 years. Comparisons between PFA and thermal energy sources, with endpoints including stroke, cognitive decline, and health‐related quality of life, would provide valuable clinical context.

## 5 Limitations

This study has several limitations. The retrospective, single‐center design may introduce selection bias and restrict the generalizability of our results. The number of procedures in patients over 75 years, while among the largest reported, remains modest and may not allow full detection of rare PFA‐specific complications. Recurrence surveillance relied on heterogeneous protocols, ranging from symptom‐triggered ECGs to short‐duration Holter monitoring; this approach likely underestimated late or asymptomatic arrhythmias, underscoring the need for longer‐term continuous rhythm monitoring in future studies. Moreover, renal function was not systematically measured in the days following the procedure and reports have linked PFA‐related hemolysis to acute kidney injury, suggesting that routine biochemical follow‐up should be considered, particularly in older adults [29].

## 6 Conclusions

Pulsed‐field ablation of AF in patients over 75 years old has a favorable safety and outcomes profile, with comparable outcomes to younger patients. Despite a higher prevalence of comorbidities and functional limitations in the elderly cohort, procedural complication rates and arrhythmia recurrence did not differ significantly from those observed in younger patients. These findings support a broader use of PFA as a rhythm control strategy in elderly patients, who have traditionally been underrepresented in clinical trials despite bearing the highest burden of AF. Further prospective studies are warranted to consolidate these observations and to explore the long‐term outcomes of PFA in older populations.

## Funding

The authors have nothing to report.

## Ethics Statement

The study complies with the reference methodology MR004 of the French National Commission for Informatics and Liberties (CNIL) and was conducted in accordance with the Declaration of Helsinki. Approval was obtained from the local ethics committee.

## Consent

All patients provided written informed consent for the anonymized use of their routine clinical data for research purposes.

## Conflicts of Interest

The authors declare no conflicts of interest.

## Data Availability

The data that support the findings of this study are available from the corresponding author upon reasonable request.
